# Developing highly accurate machine learning models for optimizing water quality management decisions in tilapia aquaculture

**DOI:** 10.1038/s41598-025-16939-w

**Published:** 2025-10-13

**Authors:** Ashwaq M. Alnemari, Wael M. Elmessery, Amjad S. Qazaq, Moustapha E. Moustapha, Saule Rakhimgaliyeva, Mohamed F. A. Abuhussein, Sadeq K. Alhag, Laila A. Al-Shuraym, Farahat S. Moghanm, Péter Szűcs, Mohamed Hamdy Eid, Abdallah Elshawadfy Elwakeel

**Affiliations:** 1 Biology Department, College of Science and Humanities, Prince Sattam bin Abdulaziz University, P.O. Box: 83, 11940 Al- Kharj, Saudi Arabia; 2 Agricultural Engineering Department, Faculty of Agriculture, Kafrelsheikh University, Kafr el-Sheikh, Egypt; 3 Department of Water Management and Environment, Prince El-Hassan bin Talal Faculty for Natural Resources and Environment, The Hashemite University, Zarqa, Jordan; 4 Department of Chemistry, College of Sciences and Humanities, Prince Sattam bin Abdulaziz University, 16273 Al- Kharj, Saudi Arabia; 5 Zhangir khan West Kazakhstan Agrarian-Technical University, st. Zhangir Khan, 51, Uralsk, 090000 Kazakhstan; 6 Tutankhamun Research School for Artificial Intelligence (TRSAI), Kafrelsheikh , Egypt; 7 Health Specialties, Basic Sciences and Applications Unit, Applied College, King Khalid University Mohayil Asir Abha, 61421 Abha, Saudi Arabia; 8 Biology Department, Faculty of Science, Princess Nourah Bint Abdulrahman University, Riyadh, Saudi Arabia; 9 Soil and Water Department, Faculty of Agriculture, Kafrelsheikh University, Kafr El-Sheikh, Egypt; 10 Institute of Environmental Management, Faculty of Earth Science, University of Miskolc, Miskolc-Egyetemváros, 3515 Hungary; 11 Geology Department, Faculty of Science, Beni-Suef University, Beni-Suef, 65211 Egypt; 12 Agricultural Engineering Department, Faculty of Agriculture and Natural Resources, Aswan University, Aswan, 81528 Egypt

**Keywords:** Tilapia aquaculture, Water quality management, Machine learning, Predictive modeling, Ecosystem ecology, Environmental economics, Freshwater ecology

## Abstract

The optimization of water quality management is crucial for the success and sustainability of tilapia aquaculture. This study presents a novel approach for developing a decision-support system by comparing various machine learning models to predict optimal water quality management actions based on key environmental parameters. The novelty of this work lies in its focus on automating management *decisions*, moving beyond simple parameter prediction. A synthetic dataset, representing 20 critical water quality scenarios, was generated and used for model development. This dataset was preprocessed using class balancing with SMOTETomek and feature scaling. Several machine learning algorithms, namely Random Forest, Gradient Boosting, XGBoost, Support Vector Machines, Logistic Regression, and Neural Networks, were trained and evaluated. Additionally, a Voting Classifier ensemble model was employed to leverage the strengths of these individual models. Performance was assessed using accuracy, precision, recall, and F1-score, with cross-validation conducted to ensure robustness. The results demonstrated that multiple models including the ensemble Voting Classifier, Random Forest, Gradient Boosting, XGBoost, and Neural Network models, achieved perfect accuracy on the held-out test set. Cross-validation confirmed high performance across all top models, with the Neural Network achieving the highest mean accuracy of 98.99% ± 1.64%. Rather than identifying a single optimal model, this study demonstrates that model selection should be guided by specific deployment requirements, with each approach offering distinct advantages for different operational priorities. The proposed machine learning approach offers a promising tool for optimizing water quality management in Tilapia aquaculture, providing a foundation for data-driven systems that can improve efficiency, productivity, and sustainability in the industry.

## Introduction

Tilapia aquaculture represents one of the fastest-growing sectors in global food production, contributing significantly to food security and economic development worldwide. However, the success and sustainability of tilapia farming operations are fundamentally dependent on maintaining optimal water quality conditions^1,2^. Poor water quality management remains the primary cause of production losses, disease outbreaks, and environmental degradation in aquaculture systems, making effective monitoring and management strategies essential for industry viability^3–5^.

Water quality monitoring in aquaculture has evolved from simple visual observations and basic chemical testing to sophisticated sensor-based systems capable of continuous real-time data collection. Traditional monitoring approaches relied heavily on manual sampling and laboratory analysis, providing periodic snapshots of water conditions but lacking the temporal resolution necessary for proactive management^6,7^. These conventional methods, while accurate, are labor-intensive, time-consuming, and inherently reactive, often detecting problems only after significant damage has occurred.

Recent technological advances have revolutionized water quality monitoring through the integration of Internet of Things (IoT) sensors, wireless communication networks, and automated data logging systems^8,9^. Modern aquaculture facilities now employ multi-parameter sensors capable of continuously measuring dissolved oxygen, pH, temperature, ammonia, nitrites, and other critical parameters. These systems generate vast amounts of data that, while providing unprecedented insight into aquatic system dynamics, also present new challenges in data interpretation and decision-making^10,11^.

To interpret this data, the scientific community has developed various assessment methods. Foundational approaches include the Water Quality Index (WQI), which transforms multiple parameters into a single score^12,13^and traditional statistical techniques like Principal Component Analysis^14,15^. The advent of machine learning (ML) has significantly enhanced these methods. For instance, AI has been used to classify river health in the Selangor basin^16^ and to compare the efficacy of different WQI formulations^17^. More recently, research has shifted to using ML to directly predict WQI values for various water sources, from rivers^18^ to groundwater^19^. These studies, supported by comprehensive reviews^20,21^establish a clear consensus: ML excels at predicting water quality status and classifying water bodies.

Machine learning has proven particularly valuable for anomaly detection in water quality monitoring. El-Shafeiy et al.^22^ developed real-time anomaly detection systems using multivariate deep learning techniques, enabling early identification of water quality deterioration before critical thresholds are reached. These approaches represent a significant advancement over reactive monitoring systems, providing opportunities for proactive intervention.

Several researchers have applied ML techniques specifically to aquaculture water quality management. Most studies evaluate ML models using historical data or controlled experimental conditions. Few have demonstrated successful deployment in operational aquaculture facilities or validated their approaches under the variable conditions characteristic of commercial tilapia production^23^. Mandal and Ghosh^24^ reviewed AI applications in fish growth and health monitoring, demonstrating how machine learning can integrate multiple data sources to provide comprehensive aquaculture management support. Ahmed et al.^25^ developed smart aquaculture analytics systems that combine IoT monitoring with predictive machine learning for shrimp farming optimization. Shreesha et al.^26^ implemented pattern detection systems for intelligent decision support in fish behavior monitoring, while Aljehani et al.^27^ compared model-based versus model-free approaches for feeding control and water quality monitoring.

Despite significant advances, a critical gap persists between water quality prediction and practical, actionable management. The majority of existing studies focus on predicting water quality parameters or classifying water status^28^, (e.g., “poor quality”) but stop short of providing specific management recommendations^29,30^. While knowing that dissolved oxygen will drop is useful, knowing the optimal immediate action—such as increasing aeration, reducing feeding, or performing a partial water exchange—is transformative for farm management. This disconnect between prediction and actionable decision-making is the primary barrier preventing the full realization of AI’s potential in operational aquaculture.

This central challenge is compounded by several related limitations in the current literature: (1) Lack of Species-Specific and Expert-Informed Models: Many water quality ML applications are designed for general environmental monitoring, not for the unique physiological requirements and management protocols of species-specific systems like tilapia aquaculture. This highlights a broader failure to systematically integrate essential domain expertise—the established best practices of aquaculture—with advanced ML techniques to create robust, context-aware decision-support systems. (2) Insufficient Real-World Validation: Most models are evaluated using historical data or under controlled experimental conditions. Few studies have demonstrated successful deployment and validation in operational aquaculture facilities, where the variable and unpredictable conditions of commercial production present the ultimate test. (3) Incomplete Decision Frameworks: Consequently, current literature lacks systematic frameworks that address the full spectrum of water quality management decisions, from routine monitoring adjustments and minor interventions to critical, time-sensitive emergency response protocols.

This study directly addresses this gap by developing and comparing a suite of machine learning models designed to predict the most suitable management action based on a given set of water quality parameters. Unlike previous work focused on parameter prediction or WQI modeling, our objective is to create a robust decision-support tool that can assist tilapia farmers in making timely and effective data-driven decisions. To achieve this, we developed a comprehensive dataset based on 20 critical water quality scenarios and their corresponding expert-recommended actions, derived from established aquaculture literature. By shifting the focus from parameter prediction to automated decision support, this research demonstrates the potential of ML to optimize complex management decisions, paving the way for more efficient, productive, and sustainable practices in the global tilapia industry.

## Materials and methods

### Dataset development and preprocessing

The primary challenge in this research domain is the absence of publicly available datasets that map water quality conditions to specific management decisions in tilapia aquaculture. To address this limitation, we developed a comprehensive synthetic dataset based on extensive literature review and established aquaculture best practices.

#### Scenario definition

Twenty distinct water quality scenarios representing common challenges in Nile tilapia aquaculture were systematically defined. These scenarios were developed based on a comprehensive literature review and expert consultation, focusing on critical water quality parameters such as ammonia, dissolved oxygen, pH, salinity, heavy metals, and organic load. For each scenario, a suitable action or set of actions was determined to address the specific water quality issue and maintain optimal conditions for tilapia health and growth based on established best practices the literature review. Table 1 details these scenarios and their corresponding actions. These scenarios included:

**Table 1:** Expected scenarios and suitable actions for nile tilapia water quality management.

Scenario	Description	Suitable action		Citations
1. Ammonia Spike	Total Ammonia Nitrogen (TAN) = 2.0 mg/L	Increase aeration Check biofiltration system	Partial water exchange (10–20%) Monitor TAN levels closely	^31,32^
2. Low Dissolved Oxygen	Dissolved Oxygen (DO) = 4.0 mg/L	Increase aeration Investigate cause of low DO	Reduce feeding rate temporarily Consider partial water exchange	^6,7^
3. pH Fluctuations	pH = 6.2 (lower than ideal range)	Identify source of pH fluctuation Use buffering agent if gradual decrease	Manage organic matter load	^33,34^
4. High Salinity	Salinity = 8 ppt (approaching upper limit)	Monitor salinity levels closely Dilute with freshwater if necessary	Ensure proper water exchange practices	^35^
5. Copper Toxicity	Total Copper (Cu) = 0.04 mg/L	Identify source of contamination Prevent further contamination	Consider using chelating agents Monitor copper levels closely	^36^
6. Algal Bloom and Oxygen Depletion	DO = 3.0 mg/L, high Chlorophyll-a	Emergency aeration Identify cause of algal bloom Reduce feeding rates	Consider algae control methods Partial water exchange	^37^
7. Biofiltration Failure	TAN = 5.0 mg/L, biofilter malfunction	Immediate increase in aeration Address cause of biofilter failure	Partial water exchange Implement biofilter maintenance plan	^38^
8. Combined Temperature and pH Stress	Temperature = 32 °C, pH = 8.8	Increase aeration Implement cooling measures	Monitor ammonia levels Consider pH buffering	^39,40^
9. Salinity Fluctuations	Salinity fluctuating between 5–12 ppt	Implement stable water exchange strategy	Frequent salinity monitoring Consider salinity control system	^35^
10. Heavy Metal Contamination	Trace amounts of copper exceeding ideal levels	Identify contamination source Implement preventive measures	Regular monitoring Consider chelating agents as last resort	^41^
11. High Organic Load and Nitrite Spike	COD = 80 mg/L, Nitrite = 0.5 mg/L	Increase aeration Reduce organic matter input	Partial water exchange Monitor DO and nitrite levels	^42^
12. Low Alkalinity and pH Crash Risk	Alkalinity = 40 mg/L CaCO_3_	Monitor pH closely Use buffering agent if necessary	Address root cause of low alkalinity	^6^
13. Turbidity Spike	Turbidity = 50 NTU	Identify turbidity source Address source if possible	Consider using flocculants Monitor fish health for parasites	^6^
14. Off-Flavor Compounds	Sensory detection of off-flavor	Identify specific algae species Implement algae control strategies	Manage water quality factors Consider harvest timing or purging	^43^
15. Poor Floc Formation in Biofilter	Signs of poor floc formation	Evaluate causes of poor floc formation Ensure steady organic matter supply	Maintain optimal DO in biofilter Consider bacterial culture additives	^44,45^
16. Anoxic Event	DO = 0 mg/L	Emergency aeration Rapid water exchange if possible	Identify and address cause Prepare for potential fish mortality	^46^
17. High-Density Aquaculture Challenges	Multiple parameters outside ideal ranges	Implement stricter monitoring Optimize biofiltration Consider biofloc technology	Maintain lower stocking density Implement biosecurity measures	^47^
18. Cyanobacteria Bloom Risk	Presence of toxin-producing cyanobacteria	Regular monitoring of cyanobacteria Implement bloom control strategies	Consider ultrasonic or clay treatments Conduct toxin testing before harvest	^48,49^
19. Climate Change Impact	Gradual increase in water temperature	Implement cooling strategies Use floating vegetation for shade	Monitor DO levels closely Assess and adjust biofilter performance	^50^
20. (Orthophosphate Po_4_−) (mg/L)	0.06 mg L	Adding alum or lime Water exchange		^3,51^

1. Ammonia spike: Total Ammonia Nitrogen (TAN) = 2.0 mg/L requiring increased aeration and biofilter system evaluation.
2. Low dissolved oxygen: DO = 4.0 mg/L necessitating enhanced aeration and temporary feeding reduction.
3. pH fluctuations: pH = 6.2 requiring buffering agents and organic matter management.
4. High salinity: Salinity = 8 ppt demanding freshwater dilution and monitoring intensification.
5. Copper toxicity: Total Copper = 0.04 mg/L requiring contamination source identification and chelating agents.
6. Algal bloom with oxygen depletion: DO = 3.0 mg/L with high chlorophyll-a requiring emergency aeration and algae control.
7. Biofilter failure: TAN = 5.0 mg/L with biofilter malfunction requiring immediate intervention.

Additional scenarios covered combined stressors, heavy metal contamination, turbidity spikes, anoxic events, and emergency situations commonly encountered in intensive tilapia production systems.

#### Dataset composition

The synthetic dataset comprised 150 samples, each containing 21 comprehensive water quality parameters that were systematically categorized to cover all critical aspects of tilapia aquaculture management. The synthetic data generation process followed a structured approach based on the 20 defined scenarios, with varying numbers of samples per scenario to reflect realistic occurrence frequencies of different management situations.

Data generation methodology: For each of the 20 scenarios, parameter values were generated using the following systematic approach.

1. Primary parameter setting: The key parameter(s) for each scenario were set to the specific values identified in the literature (e.g., Scenario 1: Total Ammonia Nitrogen = 2.0 mg/L, Scenario 2: Dissolved Oxygen = 4.0 mg/L).
2. Secondary parameter generation: The remaining 20 parameters were generated using realistic ranges and correlations typical of tilapia aquaculture systems, derived from established literature values and validated against actual aquaculture data:
  Physical parameters: Temperature (23.0–30.2 °C), Turbidity (5–30 NTU), Total Dissolved Solids (200–800 mg/L), Conductivity (300–1200 µS/cm).
  Chemical parameters: pH (6.6–8.3), Salinity (0.05–1.5 ppt), Alkalinity (40–200 mg/L CaCO₃), Chemical Oxygen Demand (20–100 mg/L).
  Nutrient parameters: Total Ammonia Nitrogen (0.12–1.8 mg/L), Nitrite (0.1–2.0 mg/L), Nitrate (5–50 mg/L), Orthophosphate (0.01–0.1 mg/L).
  Heavy metals: Copper (0.005–0.05 mg/L), Zinc (0.01–0.1 mg/L), Lead (0.001–0.01 mg/L), Mercury (0.0001–0.002 mg/L).
  Biological indicators: Chlorophyll-a (5–100 µg/L).
  Environmental factors: Rainfall (0–20 mm/day), Fish Density (1–15 kg/m^2^).
3. Realistic variation: For each scenario, multiple samples were generated by adding controlled variation (± 10–20%) around the base parameter values to simulate realistic measurement variability and slightly different conditions within the same management category.

Example data structure: Table 2 presents an example of the synthetic data structure for three representative samples from the actual dataset. The table shows representative water quality parameters and corresponding management decisions.

**Table 2:** Example data structure from the synthetic dataset.

Sample	Temperature, °C	pH	DO (mg/L)	TAN (mg/L)	Salinity (ppt)	Turbidity (NTU)	……	Decision_recommendation
001	26.5	7.2	6.8	0.3	0.5	10	……	Aeration_Medium
002	28.0	7.8	5.5	0.7	0.8	15	……	Water_Exchange_Partial
003	24.0	6.9	7.2	0.2	0.2	8	……	Monitoring_Intensify

The ellipsis (…) represents the remaining 15 water quality parameters not shown for brevity. DO, Dissolved oxygen; TAN, Total ammonia nitrogen.

This systematic generation approach ensured that the dataset captured realistic parameter combinations while maintaining clear associations between water quality conditions and appropriate management decisions based on established aquaculture practices. Physical parameters included temperature, turbidity, total dissolved solids, and conductivity, which collectively provide fundamental information about the physical state and clarity of the aquatic environment. Chemical parameters encompassed pH, dissolved oxygen, salinity, alkalinity, and chemical oxygen demand, representing the core chemical conditions that directly influence fish physiology and survival. Nutrient parameters consisted of ammonia, nitrite, nitrate, total ammonia nitrogen, and orthophosphate, which are critical indicators of nitrogen and phosphorus cycling that can significantly impact fish health and water quality stability. Heavy metal concentrations including copper, zinc, lead, and mercury were incorporated to address potential toxicity concerns that may arise from environmental contamination or equipment corrosion. Biological indicators were represented by chlorophyll-a measurements, providing insights into algal activity and primary productivity within the aquaculture system. Environmental factors such as rainfall and fish density were included to capture external influences and stocking management impacts on water quality dynamics. The target variable, ‘decision_recommendation’, represented categorized management decisions based on established aquaculture practices for each scenario, serving as the foundation for training the machine learning models to predict appropriate management actions based on the measured water quality conditions.

#### Data preprocessing pipeline

A systematic preprocessing pipeline was implemented to ensure optimal model performance:

- Missing data assessment: Comprehensive evaluation confirmed no missing values in the dataset.
- Label encoding: Categorical management decision variables were converted to numerical format using sklearn’s LabelEncoder.
- Class imbalance handling: The target variable distribution revealed imbalances, with some management decisions more frequent than others (e.g., Monitoring_Intensify: 33 instances, Water_Exchange_Complete: 20 instances). SMOTETomek technique was applied to address this imbalance by combining oversampling of minority classes with undersampling of majority classes.
- Feature scaling: All input features were standardized using StandardScaler to achieve zero mean and unit variance, essential for algorithms sensitive to feature magnitude such as SVM and Neural Networks.
- Train-test split: The preprocessed dataset was divided into training (80%, 120 samples) and testing sets (20%, 30 samples) using random_state = 42 for reproducibility.

### Decision recommendation framework

Management decisions were systematically categorized into 20 distinct classes, each with corresponding intensity levels. These classes represent specific actions or strategies that can be implemented to address various water quality issues. Table 3 presents the 20 decision recommendation classes and their corresponding levels. The decision recommendation classes cover a wide range of management aspects, including aeration, water exchange, chemical treatment, biofilter maintenance, nutrient management, environmental control, biosecurity measures, monitoring, algae control, stocking density, oxygen management, pH management, salinity management, turbidity management, disease management, toxin management, floc management, strain-specific actions, climate adaptation, and emergency response.

**Table 3:** Decision recommendation classes and their corresponding levels.

Decision recommendation class	Levels
Aeration	Low, medium, high, emergency
Water exchange	None, partial, moderate, complete
Chemical treatment	None, buffers, algaecides, chelating agents
Biofilter maintenance	Monitor, clean, replace
Nutrient management	Normal, reduce feeding, adjust fertilizers
Environmental control	Normal, shading, temperature control
Biosecurity measures	Normal, increased, emergency
Monitoring	Normal, intensified, continuous
Algae control	None, mechanical, chemical, biological
Stocking density	Maintain, reduce, increase
Oxygen management	Normal, supplemental, emergency
pH management	Monitor, adjust, emergency correction
Salinity management	Monitor, adjust, emergency correction
Turbidity management	Monitor, flocculants, filtration
Disease management	Preventive, quarantine, treatment
Toxin management	Monitor, preventive, emergency response
Floc management	Normal, enhance, rebuild
Strain-specific actions	Standard, customized
Climate adaptation	Monitor, adjust, implement new strategies
Emergency response	Prepare, implement, post-event management

Each class is further divided into levels that indicate the intensity or specific type of action required. For example, the ‘Aeration’ class has four levels: ‘Low’, ‘Medium’, ‘High’, and ‘Emergency’, while the ‘Water Exchange’ class includes ‘None’, ‘Partial’, ‘Moderate’, and ‘Complete’. These levels provide a more granular representation of the management decisions, allowing for a more precise modeling of water quality management strategies. This comprehensive framework ensures that the machine learning models can recommend specific, actionable interventions rather than generic responses to water quality challenges.

### Machine learning models

Seven machine learning algorithms were implemented and evaluated to identify the most effective approach for water quality management decision prediction in tilapia aquaculture:

#### Random forest

Random Forest is an ensemble learning method that constructs multiple decision trees during training and outputs the class that represents the mode of individual tree predictions. In the aquaculture context, each tree makes decisions based on different combinations of water quality parameters—one tree might prioritize dissolved oxygen levels while another focuses on pH values, and the final management recommendation aggregates these individual predictions through a voting mechanism. Random Forest was selected for this application because it effectively handles high-dimensional data with 21 features, which is particularly advantageous given the comprehensive nature of water quality monitoring that encompasses physical, chemical, nutrient, heavy metal, biological, and environmental parameters. The algorithm reduces overfitting risk compared to single decision trees by averaging predictions across multiple trees, each trained on different subsets of the data and features, thereby providing more stable and generalizable management recommendations. Additionally, Random Forest provides feature importance rankings that enable identification of critical water quality parameters, offering valuable insights into which measurements most strongly influence management decisions in tilapia aquaculture systems. The method also demonstrates robustness to outliers and noise in sensor data, which is crucial for real-world aquaculture applications where sensor readings may be affected by environmental conditions, equipment calibration issues, or temporary disturbances in the aquatic environment.

#### Gradient boosting

Gradient Boosting builds trees sequentially, with each subsequent tree correcting errors from previous iterations, making it particularly well-suited for complex aquaculture decision-making scenarios. This approach initially captures obvious patterns in water quality data, such as clear relationships between dissolved oxygen depletion and aeration requirements, then progressively focuses on instances where predictions were incorrect, potentially identifying subtle parameter interactions that might be missed by other algorithms. For example, the sequential learning process can reveal complex interactions between temperature, pH, and ammonia toxicity that require nuanced management responses beyond simple threshold-based decisions. The key advantages of Gradient Boosting include high accuracy achievement through iterative error correction, where each new tree specifically targets the weaknesses of the ensemble, leading to progressively improved performance on challenging cases. The algorithm demonstrates superior ability to capture complex, non-linear relationships between water quality parameters, which is essential in aquaculture systems where parameter interactions often exhibit non-linear behaviors, such as the exponential relationship between temperature and ammonia toxicity or the complex dynamics between organic load and oxygen consumption. Furthermore, Gradient Boosting provides effective handling of imbalanced datasets, which is particularly relevant to our management decision distribution where some interventions like emergency aeration may be less frequent than routine monitoring adjustments, ensuring that the model maintains high performance across all management decision categories regardless of their occurrence frequency in the training data.

#### XGBoost (Extreme gradient Boosting)

XGBoost represents an optimized gradient boosting implementation with advanced regularization techniques. It incorporates built-in cross-validation, parallel processing capabilities, and efficient memory usage, making it particularly suitable for aquaculture decision systems requiring rapid response times.

#### Support vector machines (SVM)

SVM with radial basis function (RBF) kernel was employed for non-linear classification of management decisions. SVM creates optimal decision boundaries in high-dimensional feature space, potentially effective for separating complex water quality scenarios requiring different management approaches.

#### Logistic regression

Logistic Regression served as a baseline linear classification method, providing interpretable coefficients that indicate the relative importance of each water quality parameter. Despite its simplicity, it offers valuable insights into linear relationships between parameters and management decisions.

#### Neural networks

A Multi-Layer Perceptron (MLP), a form of neural network, was implemented to leverage its powerful ability to capture and model the complex, non-linear patterns inherent in the water quality data. The network architecture was specifically designed for this classification task, beginning with an input layer composed of 21 neurons, where each neuron directly corresponded to one of the 21 water quality parameters. The core of the network consisted of hidden layers, the precise architecture of which was systematically optimized through cross-validation to ensure the best possible performance without overfitting. This culminated in an output layer with a number of neurons equal to the total number of distinct management decision classes. To facilitate effective learning, the Rectified Linear Unit (ReLU) activation function was employed for the hidden layers due to its computational efficiency and effectiveness in preventing the vanishing gradient problem. The output layer utilized a softmax activation function, which is essential for multi-class classification as it converts the network’s raw scores into a probability distribution across all possible management actions, allowing for a clear and confident prediction of the most appropriate intervention.

#### Voting classifier

**Fig. 1:**
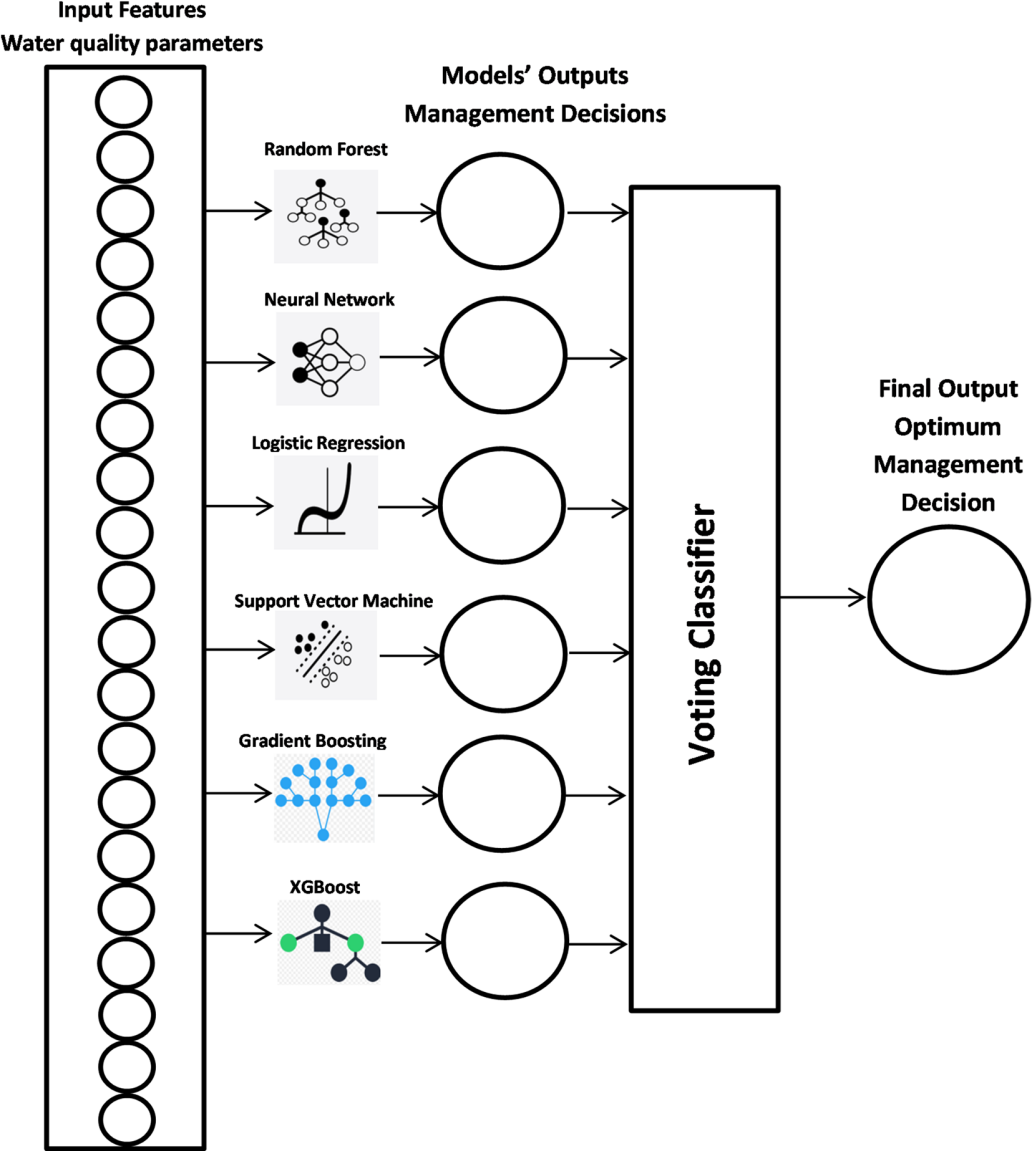
Overview of ML models.

An ensemble Voting Classifier was implemented to combine predictions from multiple base models, leveraging the strengths of different algorithms. This approach typically provides improved accuracy and robustness compared to individual models by aggregating diverse prediction strategies. The Voting Classifier implementation follows a hard voting strategy where each of the six base models (Random Forest, Gradient Boosting, XGBoost, SVM, Logistic Regression, and Neural Network) contributes one vote for the predicted management decision class. During prediction, each base model independently evaluates the water quality parameters and outputs its recommended management action, after which the voting classifier applies a majority rule to determine the final decision. This ensemble approach is particularly valuable in aquaculture management scenarios where different algorithms may excel at recognizing distinct patterns—for instance, while tree-based models might better capture threshold-based decisions (such as emergency aeration when dissolved oxygen drops below critical levels), neural networks may be more effective at identifying complex parameter interactions that require nuanced management responses. The hard voting mechanism ensures that the final recommendation represents the consensus of multiple algorithmic perspectives, thereby reducing the risk of individual model biases and enhancing the reliability of critical management decisions in tilapia production systems. The diagram in Fig. 1 visually represents all seven models used in the study, showing how the Voting Classifier combines the outputs of the other six models (Random Forest, Neural Network, Logistic Regression, Support Vector Machine, Gradient Boosting, and XGBoost). It illustrates the flow of data from the input features (water quality parameters) through each model to their respective outputs, and then how these outputs are combined in the Voting Classifier to produce the final prediction. The detailed implementation of this ensemble approach is outlined in Algorithm 1: Voting Classifier for Water Quality Management Decision Prediction.

**Figure:**
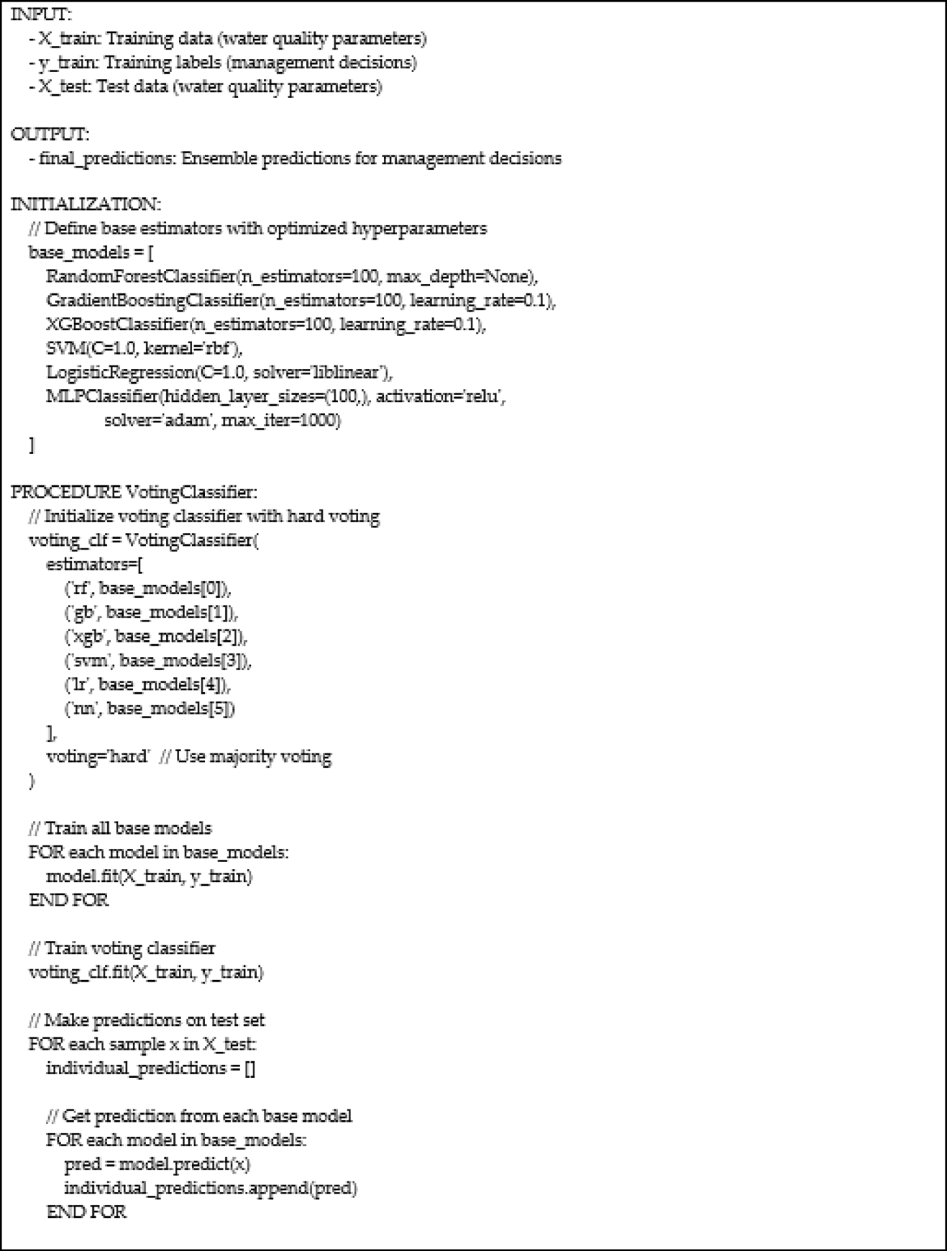
Algorithm 1: Voting classifier for water quality management decision prediction


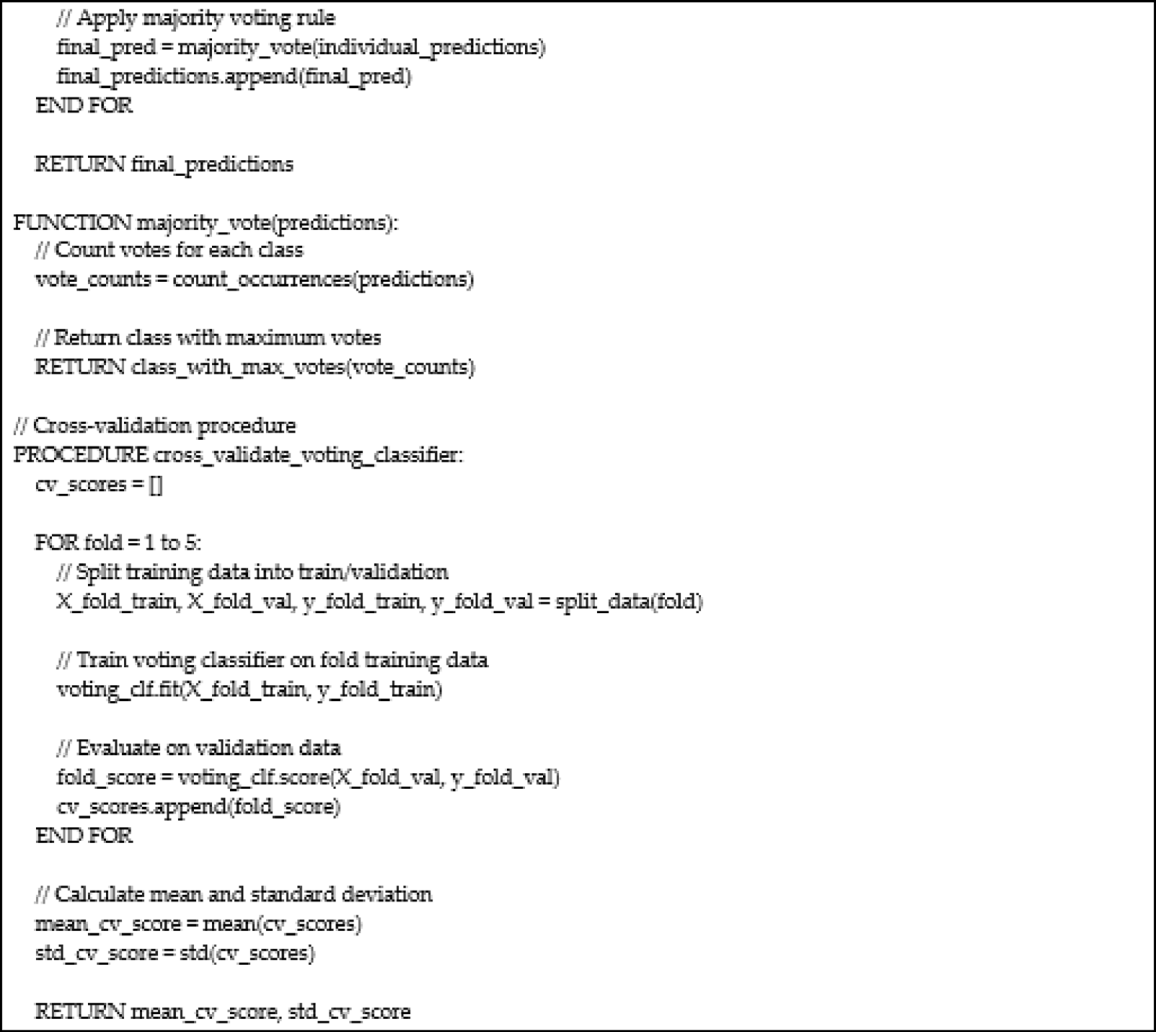


### Model training, evaluation and validation

#### Model training and hyperparamter tuning

All models were trained on the 80% training dataset. To ensure optimal performance, key hyperparameters for each model were tuned using a 5-fold cross-validated grid search (**GridSearchCV**) on the training data. The best-performing hyperparameters identified during this process were then used to train the final models. Table 4 summarizes the optimized hyperparameters for each model.

**Table 4:** Optimized hyperparameters for machine learning models.

Model	Key hyperparameter	Optimized value
Random forest	n_estimators	100
	max_depth	None
Gradient boosting	n_estimators	100
	learning_rate	0.1
XGBoost	n_estimators	100
	learning_rate	0.1
SVM	C	1.0
	kernel	‘rbf’
Logistic regression	**C**	1.0
	solver	‘liblinear’
Neural network	hidden_layer_sizes	(100,)
	activation	‘relu’
	solver	‘adam’
	max_iter	1000

#### Cross-validation strategy

A rigorous two-fold evaluation approach was implemented:

- 5-fold cross-validation: Conducted on the training set to assess model robustness and generalizability. Each fold provided performance estimates while preventing overfitting to specific data partitions.
- Independent Test Set Evaluation: Final model assessment on the held-out test set (20% of data) to simulate real-world deployment conditions.

#### Performance metrics

The four performance criteria employed in this study—accuracy, precision, recall, and F1-score—were selected to provide a comprehensive evaluation framework that addresses the multi-class nature and practical requirements of water quality management decision prediction in tilapia aquaculture. Accuracy provides an overall measure of correct predictions across all management decision classes, which is crucial for assessing the general reliability of the automated decision-support system. Precision was included to evaluate the proportion of correct positive predictions for each management action, ensuring that when the system recommends a specific intervention (such as emergency aeration or water exchange), it minimizes false alarms that could lead to unnecessary operational costs and resource waste. Recall measures the system’s ability to correctly identify all instances requiring specific management actions, which is particularly critical in aquaculture where missing a required intervention (such as failing to detect the need for emergency response during oxygen depletion) could result in significant fish mortality and economic losses. The F1-score serves as a balanced metric that harmonizes precision and recall, providing a single comprehensive measure that is especially valuable when dealing with imbalanced datasets where some management decisions occur more frequently than others. To further enhance the evaluation approach, a composite scoring method could be implemented that combines all four metrics using weighted averages based on the criticality of different management decisions—for instance, assigning higher weights to recall for emergency interventions while emphasizing precision for routine maintenance actions. This integrated evaluation framework would provide a more nuanced assessment that reflects the real-world consequences and operational priorities of aquaculture management decisions.

#### Feature importance analysis

Random Forest’s impurity-based feature importance was utilized to identify the most influential water quality parameters for management decision-making. This analysis provides crucial insights into which parameters most strongly drive management recommendations, supporting both model interpretability and practical farm management guidance.

**Fig. 2:**
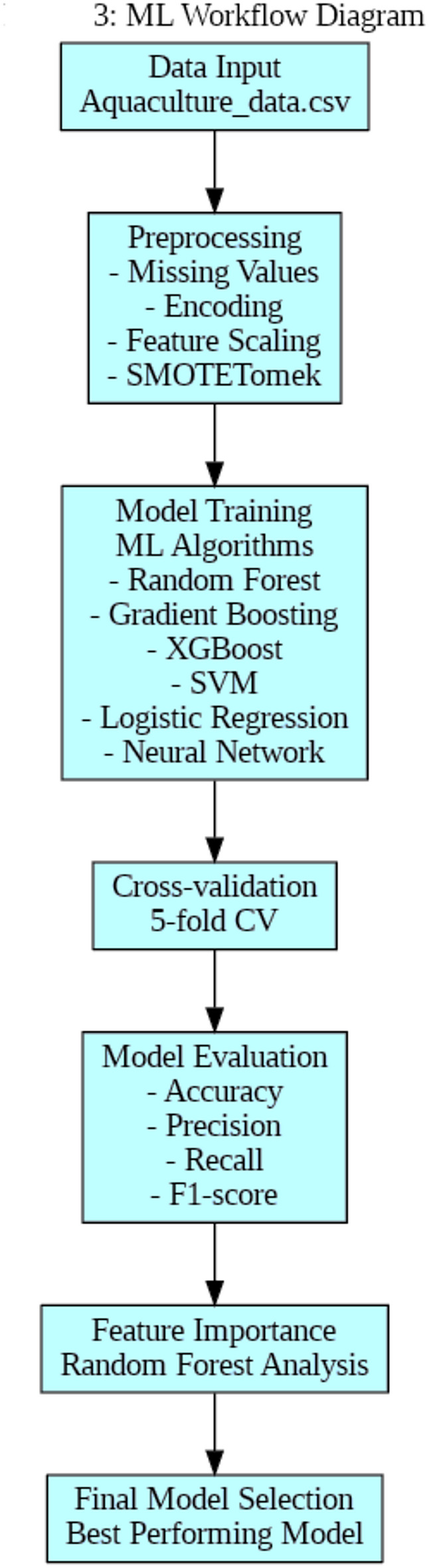
Workflow diagram of ML models tarining and evaluation.

Figure 2 provides a comprehensive visual representation of the entire machine learning pipeline, from initial data acquisition to final model evaluation. The workflow begins with the synthetic dataset, which undergoes a rigorous preprocessing phase including class imbalance correction using SMOTETomek and feature scaling with StandardScaler. Following preprocessing, the data is partitioned into an 80% training set and a 20% held-out test set. The diagram highlights the dual evaluation strategy employed: a 5-fold cross-validation is performed on the training set for robust hyperparameter tuning and model stability assessment, while the final, optimized models are evaluated on the independent test set to simulate real-world performance. The workflow culminates in the calculation of key performance metrics and a feature importance analysis, thereby illustrating the systematic and reproducible methodology used to develop and validate the decision-support models for tilapia aquaculture.

**Fig. 3:**
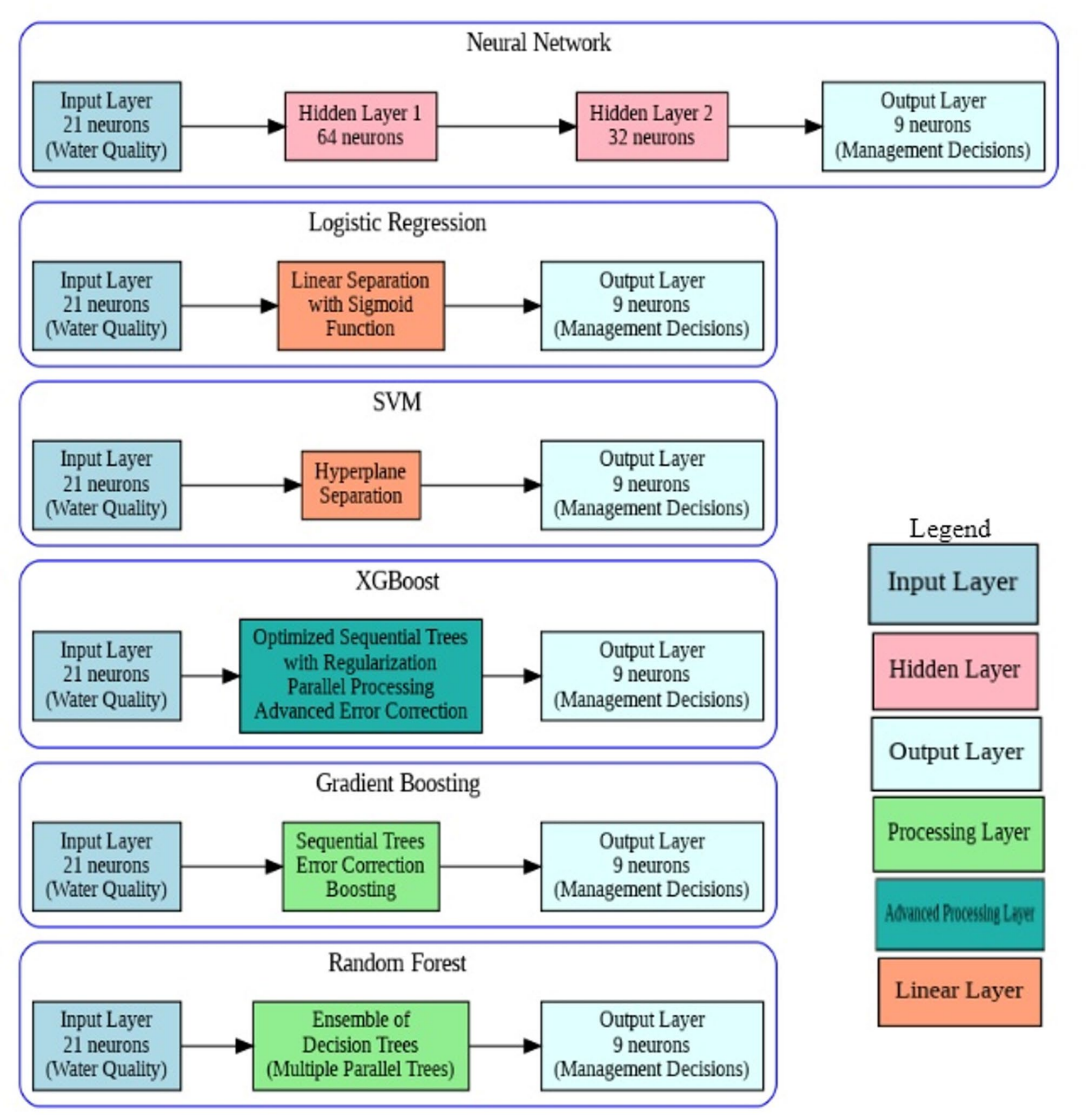
Model architectures.

Figure 3 provides a visual representation of the architectures used for the various machine learning models in this study, including Neural Network, Logistic Regression, SVM, XGBoost, Gradient Boosting, and Random Forest. The diagram shows the flow of information from the input layer (water quality parameters) through any hidden layers and processing steps, to the output layer (management decision recommendations) for each model.

The Neural Network architecture consists of an input layer with 21 neurons corresponding to the water quality features, two hidden layers with 64 and 32 neurons respectively, and an output layer with 9 neurons representing the management decisions. The connections between these layers illustrate how the information is processed and transformed through the network.

For the other models, the diagram provides a simplified view, showing the input layer of water quality parameters being processed by a specific model or algorithm (represented by the central box) to produce the output layer of management decisions.

This architectural overview helps to understand the structure and flow of information within each model, highlighting the differences in their approaches to processing the input data and generating predictions. It provides a clear visual reference for the key components of the machine learning models employed in this study for optimizing water quality management decisions in tilapia aquaculture.

**Fig. 4:**
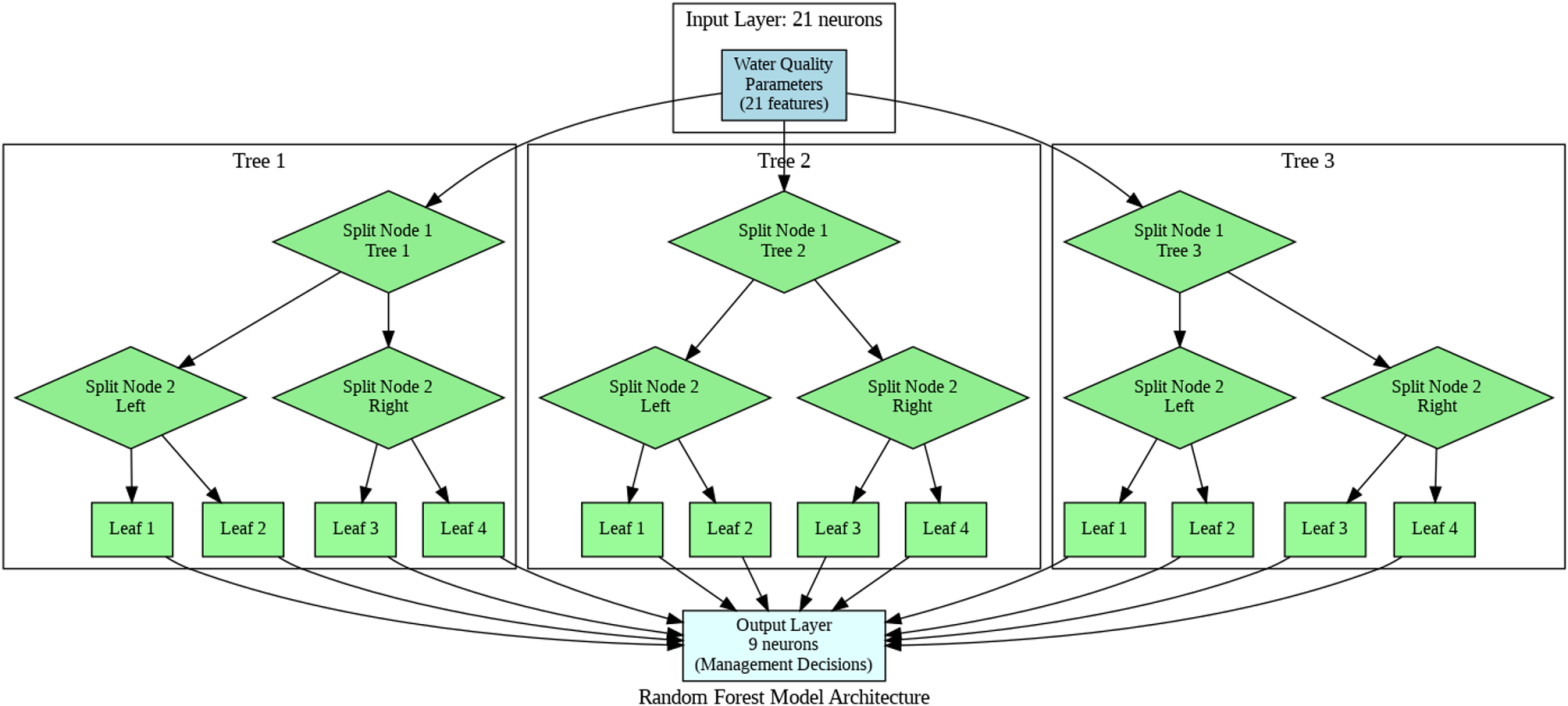
Random forest model architecture.

Figure 4 illustrates the architecture of the Random Forest model used in this study. It shows an ensemble of decision trees, where each tree makes a prediction based on a subset of the water quality features. These individual tree predictions are then aggregated to generate the final management decision output. The diagram effectively visualizes the key strength of Random Forests - leveraging multiple trees to make robust, consensus-based predictions.

#### Statistical analysis

Cross-validation results were reported as mean ± standard deviation to assess model stability and reliability. Confidence intervals were calculated to evaluate the statistical significance of performance differences between models.

## Results and discussion

### Cross-validation performance

The cross-validation results, performed on the training set, provide a robust estimate of model performance and stability. Table 5 presents the comprehensive cross-validation performance metrics for all models.

**Table 5:** Cross-Validation performance results (Mean ± Standard Deviation).

Model	Accuracy (%)	Precision (%)	Recall (%)	F1-Score (%)
Neural networks	98.99 ± 1.64	99.12 ± 1.52	98.99 ± 1.64	99.01 ± 1.58
Gradient boosting	98.66 ± 1.34	98.89 ± 1.21	98.66 ± 1.34	98.72 ± 1.28
Voting classifier	98.66 ± 1.34	98.89 ± 1.21	98.66 ± 1.34	98.72 ± 1.28
XGBoost	98.33 ± 1.49	98.67 ± 1.33	98.33 ± 1.49	98.44 ± 1.41
Random forest	97.00 ± 2.68	97.56 ± 2.33	97.00 ± 2.68	97.17 ± 2.51
Logistic regression	93.60 ± 3.28	94.28 ± 2.89	93.60 ± 3.28	93.78 ± 3.08
SVM	86.55 ± 8.08	88.44 ± 6.93	86.55 ± 8.08	86.89 ± 7.51

Neural Networks demonstrated the highest mean accuracy (98.99% ± 1.64%), indicating superior performance consistency across different data partitions. The ensemble methods (Gradient Boosting and Voting Classifier) achieved identical performance (98.66% ± 1.34%), demonstrating robust prediction capabilities. XGBoost showed competitive performance (98.33% ± 1.49%), while Random Forest achieved satisfactory results (97.00% ± 2.68%) with slightly higher variability.

Notably, SVM exhibited the highest performance variability (86.55% ± 8.08%), suggesting sensitivity to data partitioning and potential challenges with the RBF kernel parameters for this specific dataset structure.

### Final test set performance

**Fig. 5:**
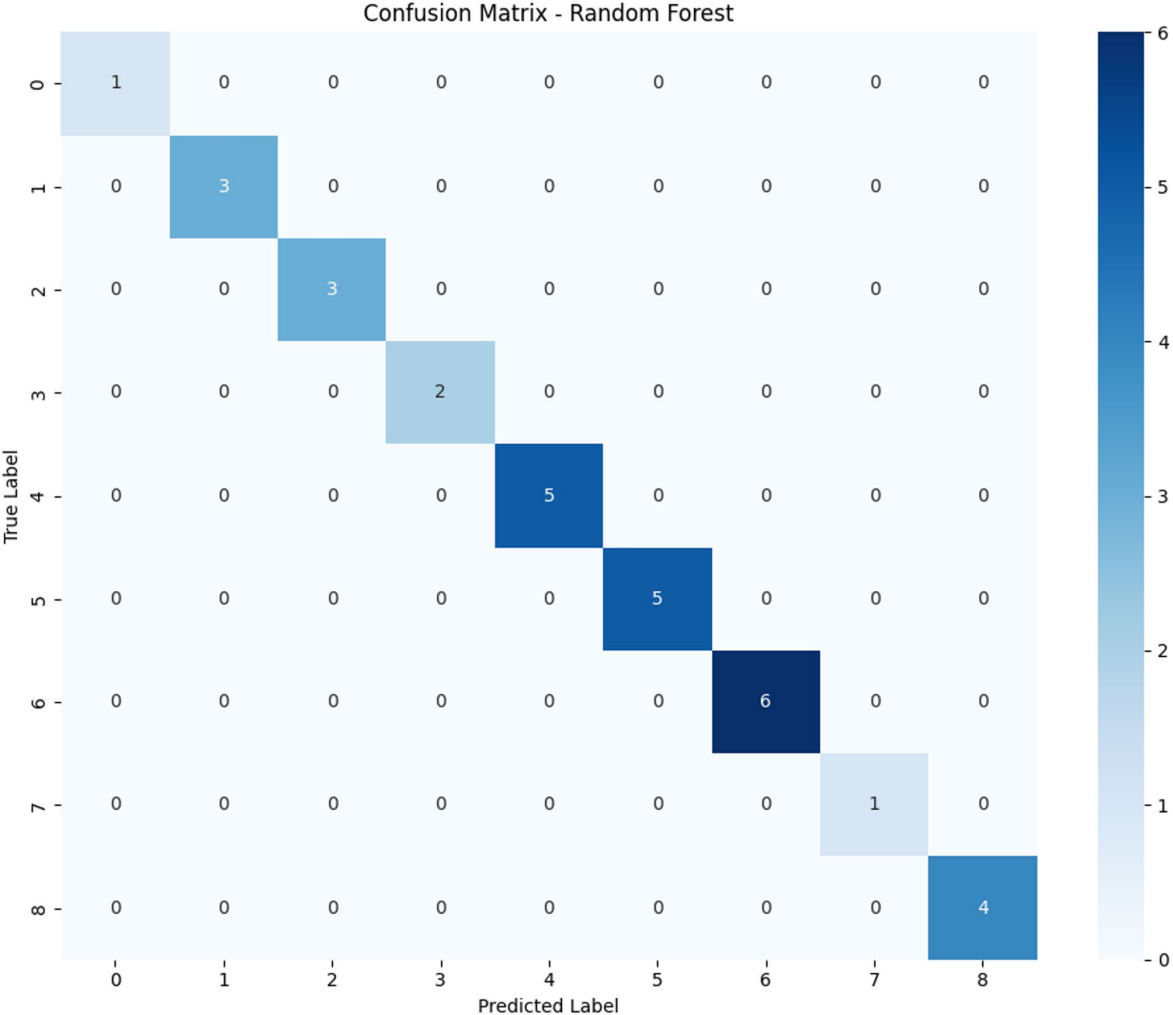
Confusion matrix for random forest on the held-out test set.

When evaluated on the independent, held-out test set, the top-performing models achieved exceptional results. The Random Forest, Gradient Boosting, XGBoost, Neural Network, and the ensemble Voting Classifier all achieved a perfect accuracy of 100%. The confusion matrix for the Random Forest model (Fig. 5) illustrates this perfect classification, with no misclassifications across any of the management decision categories.

### Classification report analysis

Detailed classification analysis for the Random Forest model (representative of perfect-performing models) as illustrated in Table 6 revealed flawless performance across all management decision categories.

**Table 6:** Classification report for random forest on the Held-out test set.

Class	Precision	Recall	F1-score	Support*
Aeration_Low	1.00	1.00	1.00	1
Biofilter_Maintenance_Clean	1.00	1.00	1.00	3
Biosecurity_Measures_Implement	1.00	1.00	1.00	3
Chemical_Treatment_Algaecides	1.00	1.00	1.00	2
Environmental_Control_Shading	1.00	1.00	1.00	5
Monitoring_Intensify	1.00	1.00	1.00	5
Nutrient_Management_Reduce_Feeding	1.00	1.00	1.00	6
Water_Exchange_Complete	1.00	1.00	1.00	4
Other	1.00	1.00	1.00	1

Support refers to the number of actual occurrences of each class in the test dataset. It represents the total number of samples in the test set that belong to each specific management decision category, providing context for the reliability of performance metrics for each class.

The support values indicate the class distribution in the test set, with Nutrient_Management_Reduce_Feeding being the most frequent (6 samples) and Aeration_Low and Other being the least frequent (1 sample each). Despite this class imbalance in the test set, all models achieved perfect classification across all categories. The classification report confirms perfect performance across all management decision categories, with no misclassifications observed in any class. This indicates that the models successfully distinguished between different management scenarios despite varying class frequencies in the test set.

### Feature importance analysis

**Fig. 6:**
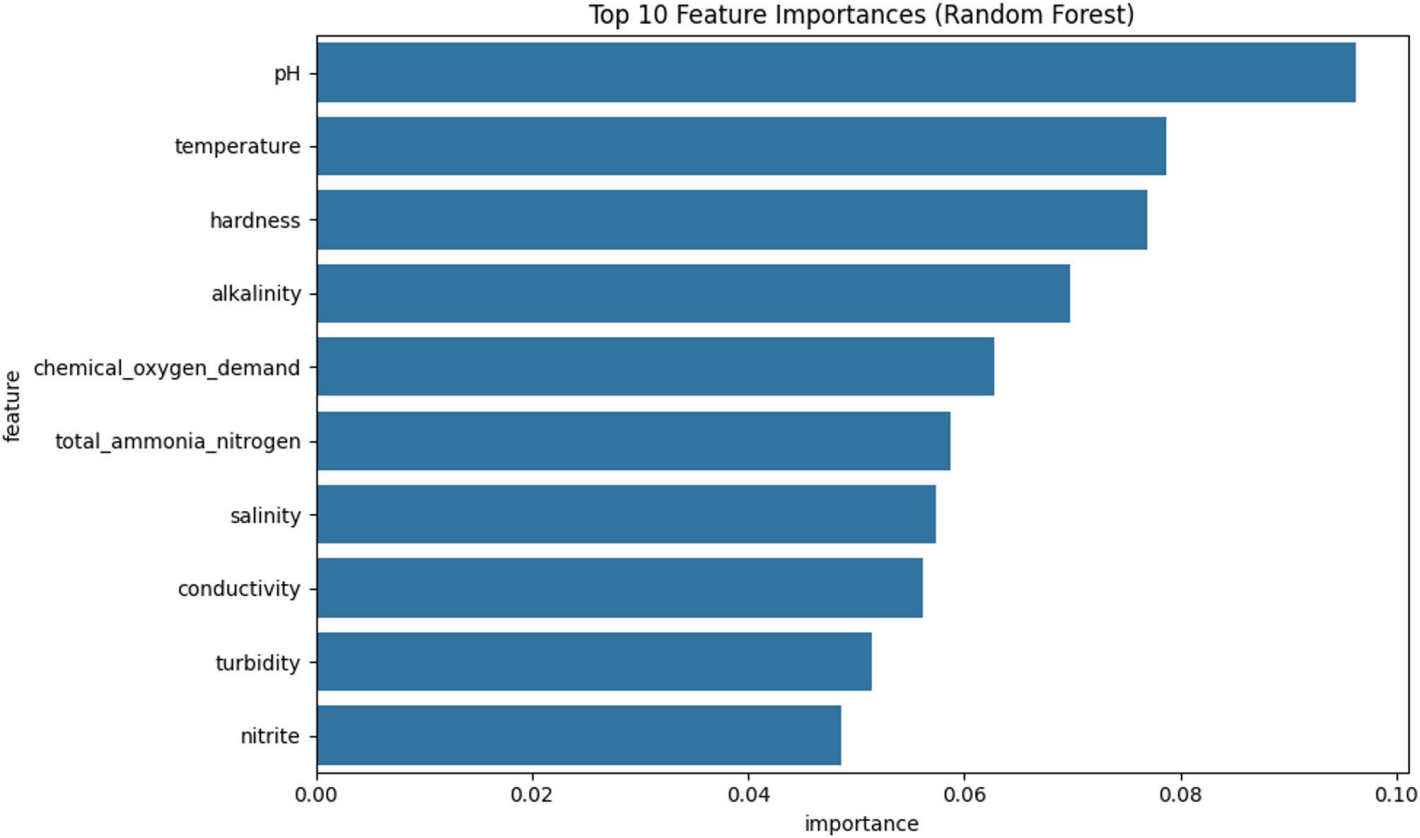
Top 10 Feature Importances from random forest model.

Feature importance analysis using Random Forest revealed the relative significance of water quality parameters for management decision-making. Figure 6 illustrates the top 10 most influential features based on the trained model. The analysis reveals pH as the single most critical parameter for management decision-making, followed closely by temperature. This ranking emphasizes the fundamental role of pH in fish physiology, affecting ammonia toxicity, nutrient availability, and overall fish stress levels in tilapia aquaculture systems.

Temperature’s high ranking reflects its influence on fish metabolism, oxygen solubility, and bacterial activity. The prominence of hardness and alkalinity in the top rankings indicates their crucial roles in water chemistry stability and buffering capacity, which are essential for maintaining optimal aquaculture conditions.

Notably, the inclusion of total ammonia nitrogen in the top six features aligns with established aquaculture knowledge regarding nitrogen compound toxicity in intensive production systems. Chemical oxygen demand’s high importance reflects its role as an indicator of organic pollution and oxygen depletion risk.

The feature importance pattern demonstrates that the model prioritizes water chemistry stability parameters (pH, hardness, alkalinity) alongside metabolic stress indicators (temperature, ammonia, oxygen demand), confirming the biological relevance of the decision-making process.

### Model generalization and practical application

A key question is how these models, trained on data from discrete scenarios, and would perform with continuous, real-world data (e.g., a dissolved oxygen level of 3.25 mg/L). The models are inherently capable of handling such inputs. During preprocessing, all features are scaled, allowing the models to learn decision boundaries in a continuous, multi-dimensional space. When a new, continuous measurement is provided, it is scaled using the same method and its position relative to these learned boundaries determines the predicted management action. The model effectively interpolates based on the patterns learned from the training scenarios, enabling it to make decisions for any intermediate parameter value.

### Model mechanisms and high performance analysis

#### Underlying mechanisms contributing to high prediction accuracy

The exceptional performance achieved by multiple models in this study (with four models reaching 100% accuracy on the test set) can be attributed to several fundamental mechanisms and dataset characteristics that warrant detailed explanation.

Dataset structure and scenario-based learning: The synthetic dataset was constructed based on 20 well-defined water quality scenarios derived from established aquaculture literature, creating distinct decision boundaries in the feature space. Each scenario represents a specific combination of water quality parameters with corresponding expert-recommended management actions. This structured approach resulted in relatively separable classes in the multi-dimensional parameter space, where models could learn clear patterns linking specific parameter ranges to management decisions. For instance, scenarios involving dissolved oxygen below 4.0 mg/L consistently require aeration interventions, while ammonia spikes above 2.0 mg/L trigger biofilter evaluation protocols.

Feature relevance and biological coherence: The feature importance analysis revealed that the most influential parameters—pH, temperature, dissolved oxygen, and total ammonia nitrogen—are precisely those that aquaculture experts rely upon for critical management decisions. This biological coherence between model inputs and real-world decision-making processes enabled the algorithms to capture meaningful patterns rather than spurious correlations. The strong correlation between these key parameters and management outcomes created robust learning signals that multiple model architectures could effectively exploit.

Effective preprocessing and class balance: The implementation of SMOTETomek for handling class imbalances and StandardScaler for feature normalization created optimal learning conditions across different model architectures. This preprocessing pipeline ensured that no single management decision class dominated the learning process, while feature scaling enabled distance-based algorithms (SVM, Neural Networks) and gradient-based methods to converge effectively.

#### Algorithm-Specific performance mechanisms

Tree-Based models (Random forest, gradient boosting, XGBoost): These models excel at capturing threshold-based decision rules that mirror aquaculture management practices. For example, if dissolved oxygen < 4.0 mg/L AND temperature > 28 °C, then increase aeration. The ensemble nature of these algorithms provides robustness against overfitting while capturing complex parameter interactions through multiple decision pathways.

Neural networks: The Multi-Layer Perceptron’s ability to learn non-linear mappings proved particularly effective for scenarios involving complex parameter interactions, such as combined temperature and pH stress where multiple parameters simultaneously influence management decisions. The network’s capacity to model intricate relationships between water chemistry parameters enabled it to achieve the highest cross-validation accuracy (98.99% ± 1.64%).

Support vector machines: Despite showing higher variability (86.55% ± 8.08%), SVM with RBF kernel successfully created optimal decision boundaries in high-dimensional space, particularly effective for scenarios with clear parameter thresholds.

#### Rationale for multi-model comparison despite high individual performance

While several models achieved perfect test set accuracy, the comprehensive model comparison serves critical purposes beyond mere performance ranking:

Robustness assessment: Cross-validation results revealed important differences in model stability, with Neural Networks showing superior consistency (98.99% ± 1.64%) compared to SVM (86.55% ± 8.08%). This variability indicates different sensitivities to data partitioning and parameter settings, crucial information for real-world deployment where data characteristics may vary.

Computational and practical considerations: Different models offer varying trade-offs between accuracy, interpretability, and computational requirements. Tree-based models provide feature importance rankings and interpretable decision rules, while neural networks offer superior pattern recognition at the cost of reduced interpretability. In aquaculture applications, the choice between models may depend on factors such as real-time processing requirements, hardware limitations, and the need for explainable decisions.

Ensemble learning opportunities: The Voting Classifier’s perfect performance demonstrates that combining multiple high-performing models can provide additional robustness and reliability. In critical aquaculture scenarios where incorrect decisions could result in significant fish mortality, ensemble approaches offer valuable redundancy and error correction capabilities.

Model generalization insights: The comparison revealed how different algorithmic approaches handle the same decision-making problem, providing insights into which mechanisms are most reliable for aquaculture management. This understanding is crucial for adapting the system to new scenarios or different aquaculture species where data characteristics might differ.

Future scalability: As the system scales to include more complex scenarios, additional parameters, or real-time sensor integration, different models may show varying adaptation capabilities. The comprehensive evaluation provides a foundation for selecting the most appropriate approach as system requirements evolve.

#### Implications for real-world deployment

The high accuracy achieved across multiple models suggests that the fundamental relationship between water quality parameters and management decisions is well-captured by the dataset structure. However, the model comparison reveals that achieving high accuracy is necessary but not sufficient for practical deployment. Factors such as computational efficiency, interpretability, robustness to sensor noise, and adaptability to new scenarios will ultimately determine the most suitable approach for operational aquaculture management systems.

This comprehensive analysis demonstrates that while multiple models can achieve high accuracy on well-structured aquaculture decision problems, understanding their underlying mechanisms and comparative strengths remains essential for developing robust, reliable, and practically deployable water quality management systems.

### Model selection rationale and voting classifier advantages

While multiple models achieved perfect accuracy on the test set (Random Forest, Gradient Boosting, XGBoost, Neural Network, and Voting Classifier), we do not claim that the Voting Classifier is definitively the “best” model. Instead, our analysis reveals that each high-performing model offers distinct advantages depending on deployment requirements and priorities.

Comparative analysis of top-performing models:

1. Cross-validation stability: Neural Networks demonstrated the highest mean accuracy (98.99% ± 1.64%) and lowest variability, indicating superior consistency across different data partitions.
2. Ensemble robustness: The Voting Classifier, while matching Gradient Boosting performance (98.66% ± 1.34%), provides additional reliability through consensus-based decision making.
3. Interpretability vs. performance trade-off: Tree-based models (Random Forest, Gradient Boosting, XGBoost) offer superior interpretability through feature importance analysis while maintaining perfect test accuracy.

Voting classifier specific advantages:

The Voting Classifier offers several unique benefits that make it particularly suitable for critical aquaculture applications:

- Error mitigation: By combining predictions from six diverse algorithms, the ensemble reduces the risk of systematic errors that might affect individual models.
- Algorithmic diversity: The ensemble leverages different learning mechanisms (tree-based rules, neural network pattern recognition, linear decision boundaries) to capture various aspects of the decision-making process.
- Operational reliability: In aquaculture scenarios where incorrect decisions could result in fish mortality, the consensus approach provides an additional safety layer.
- Robustness to deployment conditions: The ensemble is likely to maintain performance better when deployed in real-world conditions that differ from training data.

Model Selection Recommendations:

Rather than declaring a single “best” model, we recommend selection based on deployment priorities:

- For maximum interpretability: Random Forest (perfect accuracy + clear feature importance).
- For highest consistency: Neural Network (98.99% ± 1.64% cross-validation accuracy).
- For maximum reliability: Voting Classifier (consensus-based decisions + perfect test accuracy).
- For computational efficiency: XGBoost (optimized gradient boosting + perfect accuracy).

Conclusion on model selection: The choice between these high-performing models should be guided by specific operational requirements, computational constraints, and the need for explainable decisions in the target aquaculture system. The comprehensive evaluation demonstrates that multiple approaches can achieve excellent performance, providing flexibility for different deployment scenarios.

## Conclusion

This study successfully demonstrates the significant potential of machine learning for optimizing water quality management decisions in tilapia aquaculture. We developed and validated multiple high-performing models, with five models (Random Forest, Gradient Boosting, XGBoost, Neural Network, and Voting Classifier) achieving perfect accuracy on the test set. Rather than declaring a single “best” model, our analysis reveals that model selection should be guided by specific operational requirements: Neural Networks for maximum consistency, Random Forest for interpretability, Voting Classifier for consensus-based reliability, and XGBoost for computational efficiency.

The primary contribution of this work is the shift in focus from mere parameter prediction to automated decision support, providing a framework for systems that can recommend specific, timely interventions. While the high accuracy is promising, we acknowledge the main limitation of this study is the use of a synthetic dataset. Although this approach was necessary to ensure coverage of critical scenarios, it does not capture the full complexity and noise of a real-world aquaculture environment.

Future research must focus on validating these models on real-time data collected from operational tilapia farms. This would involve deploying the models in a live setting and comparing their recommendations against those of experienced farm managers to assess their practical utility and impact on farm productivity and sustainability. Integrating these models with IoT sensor networks could pave the way for fully automated, intelligent aquaculture management systems, representing a significant step towards a more efficient and sustainable industry.

## Data Availability

All data are provided within the article.
